# The Problem of Observing Sociotechnical Entities in Social Science and Humanities Energy Transition Research

**DOI:** 10.3389/fsoc.2021.699362

**Published:** 2022-02-04

**Authors:** Christian Büscher

**Affiliations:** Karlsruhe Institute of Technology (KIT), Karlsruhe, Germany

**Keywords:** loose and tight couplings, social systems, technology, control, change, actionability, complexity, nontransparency

## Abstract

The notion of “sociotechnical” is an important concept for interdisciplinary research on the transformation of the energy supply. Different branches of research agree that the provision, transmission, and distribution of energy are not simply a matter of physics. The transformation of the energy infrastructure is significantly a societal project, carried by technical innovation *and* social change. However, in social science and humanities research the interrelation between technical and social processes is often not explicitly explored, even though the interrelationship is the decisive descriptor that distinguishes sociotechnical entities from their environment. This article examines the merits of enriching the concept of sociotechnical by adding the distinction between *tight* and *loose couplings* in technical operations and human activities. While tight couplings are necessary to sustain control, they hamper change, and while loose couplings are necessary to adapt and to uphold choice, they increase complexity. Additionally, the article concludes that the introduction of “smart” technologies—an essential vision of the energy transformation—changes the composition of tight and loose couplings. Technical ideas such as machine learning and artificial intelligence go beyond mere automation. We might as well face a new sociotechnical reality. The introduction of intelligence in systems makes more loose couplings necessary. Paradoxically, this allows for new functionality and services by establishing complex operations while at the same time diminishing control by social systems.

## Introduction

In recent years, the phenomenon of the *energy transition* has become a focal point for social science and humanities (SSH) research.^1^ Such transitions concern societal systems and large infrastructures, including processes where technical and nontechnical developments interact. Researchers, therefore, opt to analyze such entities in an integrative manner. To this end, a variety of studies have used the concept of “sociotechnical systems,” for example, in research on the history of electrification (Hughes 1983; Hughes 1986), on the governance of infrastructures (Mayntz 2009, 122), and on transition processes (Geels 2004; Miller, Iles, and Jones 2013; Bolton and Foxon 2015). One of the main achievements of research on sociotechnical systems is to expose the role that social arrangements play in the emergence, development, and operation of large technical systems. Yet, the interrelation between technical and social elements of such systems is not always explicitly discussed and rather taken for granted.

The common denominator for observing sociotechnical entities is the assumption that they comprise more than the artificial, material elements usually ascribed to technical systems. Sociotechnical systems are therefore described as “hybrid systems” (Franssen and Kroes 2009, 223). In this sense, the label sociotechnical is used in a generalized way, assuming that heterogeneous elements are relevant for the functioning of such systems and, most importantly, equally heterogeneous interrelations between these elements constitute an emergent type of entity.

Thus, to grasp the nature of sociotechnical entities scholars have chosen different concepts such as systems, networks, or constellations as a means of observation. First use of the term sociotechnical is attributed to the work of the Tavistock Institute, which brought attention to the relationship between manual labor and technical operation and to the consequences of changes in one or the other on the organization of industrial production (Trist and Bamforth 1951; Herbst 1974). The notion of sociotechnical is also notoriously attributed to the works of Thomas Hughes, although he actually uses it scarcely in his major work “Networks of Power”.^2^ When he uses it, Hughes wanted to extend the scope of investigation beyond technical manifestations to include social ones, i.e., the relevant structures and institutions, into the observation of large technical systems. On the other hand, Bruno Latour is often cited as a proponent of sociotechnical concepts. He, however, does not use this term in his depiction of the actor-network theory (ANT) at all. Instead, he treats everyone (humans) and everything (artefacts) as an “actant,” all of which interact in a network without clear boundaries (Callon 1987; Latour 1988; Latour et al., 2005). Latour and Callon, therefore, emphasize the relevance of the technical artefact in the observation of the social. However, with ANT the concept of sociotechnical *systems* dissolves in front of our eyes, because networks hardly constitute boundaries (Baecker, 2011, 21f). These prominent examples showcase how scholars modify their observation with the use of the concept sociotechnical: to observe relations, to extent boundaries, or to dissolve boundaries altogether.

Despite the convoluted career of the notion “sociotechnical,” it has been picked-up by various branches of STS research, experiencing a renaissance in innovation system research and system transition research.^3^ Here again, conceptual problems arise when scholars try to depict the entity of a specific research object. All in all, there is not much appetite to define sociotechnical systems in a strict manner such as in classical systems theory (clear system/environment distinctions, elements, relations, boundary control). In their seminal work about technological change, Rip and Kemp forgo formulating a definition of a strict system concept and instead opt for the idea of a constellation of heterogeneous components (Rip and Kemp 1998, 330). They emphasize the *embedded nature* of technology in social realities and anticipate the conceptual perils of employing a system-environment denotation: “[…] configurations that work cannot be demarcated from the rest of society in a simple and obvious way” (Rip and Kemp 1998, 331). This leads to the conclusion, that an inventory-based treatment of the energy system with the aim of providing a form of objective completeness is neither possible nor meaningful. Also, drawing strict boundaries is more an empirical, case-to-case matter than a question of theoretical definitions (Geels 2004, 901).^4^ In many writings, these perils are circumvented by using the term “system” in a rather unspecific, metaphorical manner: *“The energy system as a complex societal system can be defined as all those actors and artifacts that together produce the societal function energy”* (Verbong and Loorbach 2012, 9). In this depiction, the entity in question is defined as a complex that gains and maintains its unity as a result of a common function. Others abandon the term system altogether and, instead, choose alternative notions such as sociotechnical regimes, configurations, or constellations. In some cases, all these terms are used in an arbitrary manner.

Nonetheless, most writers refer to an object of change, which is construed essentially as an entity made up of sociotechnical elements and relations. Thus, the instructiveness of the term can therefore not be found in the unity insinuated by the term system, network, or constellation, but rather in the relations of socio and technical described as “aligned” or “embedded.” These terms themselves are also rather fuzzy and noninstructive. It is therefore worth looking for more precise terms. In the following, I want to introduce the distinction between *tight* and *loose* coupling, in order to elaborate on the determining or conditioning relations between technical and social elements. “Elements” refers to events that are brought about in the course of technical and social operations, and which constitute, sustain, reproduce, and change structures and institutions.^5^ While technical operations function by employing physical, biological, or chemical causalities (dominantly deterministic relations), social operations interlink social actions as communication (dominantly selective, rather conditioning relations). In this way, the concept of sociotechnical can be clarified by emphasizing the contrast between technology and some traits of social realities. This should help to clarify how both align to form a sociotechnical unity. We can determine how tight and loose couplings of events are always in effect to various degrees in sociotechnical entities, and how the different composition of tight and loose coupling solves functional problems and brings about new ones (*Tight and Loose Coupling in Sociotechnical Relations*).

I will then discuss how different approaches in SSH observe their object of research guided by this distinction. In the first case, research emphasizes tight couplings and the reduction in loose couplings with regard to the problem of control. To manage this problem it is essential to align occurrences in moment-to-moment operation in order to produce a service or an output (*Structures of Control in Sociotechnical Operation*). The other, most recent source of interest in contrast emphasizes the creation and exploitation of loose couplings in order to facilitate change and system transitions, as some technical paradigms (manifesting tight couplings) are considered detrimental to sustainable development and are supposed to be replaced. This problem refers essentially to the coevolution of social and technical elements that bring about and sustain the entities in focus (*Change in and of Sociotechnical Entities*). Finally, the discussion will be steered in the direction of how new technical developments introduce novel loose couplings into the realm of sociotechnical relations (e.g., smart tech, AI). As I like to argue, this poses a development which calls for new social arrangements (e.g., system trust) in order for it to function as intended (*Rearranging Tight and Loose Couplings in Digital Sociotechnical Realities*). I conclude with a summary, with the objective of better understanding the benefit of using the term sociotechnical (systems, configurations, constellations, etc.) in energy transitions (*Conclusion*).

## Tight and Loose Coupling in Sociotechnical Relations

The concept of coupling itself refers to the interlinkage of elements and to the complexity of systems.^6^ The assumption of coupling rejects any complete independence of elements; however, it does not always imply cause-effect relations. Only if we characterize couplings as strict or rigid to they represent more than a correlation. Couplings always limit the range of possibilities for how elements are related to each other. We can find the idea of coupling in cybernetics, where the observation of tight couplings serves as a basis for analytical determination and, thus, for eliminating chaos, chance, and contingency. Loose coupling of elements leads to the *eigenbehavior* of systems (von Foerster, 1972). Similar ideas can be found in organization theory. Management—the technique of organizing—attempts to enforce rigid and therefore predictable behavior in distinction to loose behavior (March and Simon 1993, 56ff.). or in other words, exploiting loose couplings addresses the issue of rationality despite indeterminacy (Weick 1976; Orton and Weick 1990). In eco system science, loose and rigid couplings (connectedness) are analyzed in terms of the resilience of natural systems interacting with human systems (Holling and Gunderson 2002). Finally, we can draw inspiration from theories of media and form. There, strict couplings address the problem of complexity in the determination of a form as a “bounded combinatory capability” out of various mediated possibilities like in the technique of language use (Luhmann 1995, 160).

Also, in order to grasp the meaning of a hybrid concept like “sociotechnical,” it is useful to concentrate first on the singular parts of the composition. It is now generally accepted, that the concept of technology includes not only physical installations, but also the knowledge, practices, skills, and techniques to achieve goals or to serve a purpose (Dafoe 2015, 1051). Nonetheless, the defining criterion of technology cannot be the achievement of specific goals and fulfillment of a purpose. Goals and purposes are themselves constantly changing concepts, open for interpretation and conflict, in essence, *contingent*.^7^ That being said, technology designates a specific achievement, namely the successful isolation of strict cause-and-effect relations from all the other cause-and-effect relations in effect in a system’s surroundings. In order to work as intended, technology must achieve a rigor simplification.^8^ Nonetheless, simplification does not lead to “simplicity.” It leads to reduced complexity in the sense of reducing the number and variety of possible relationships between elements. With technology this is achieved with a *tight coupling* of elements (Luhmann, 2012, 317).

Functioning technology is measured by the successful isolation of specific cause-and-effect relationships. Only then it is possible to maintain stable expectations about the possible outputs of systems: “The rationality of technology aims at the *reduction of contingency and uncertainty via* the mastery of instrumentalities and time through planning and ordering” (Simpson, 2009, 191). Through the elimination of external interference, processes become more controllable, resources more projectable, and faults both recognizable and attributable. In a very abstract way, technology is conceptualized as “functioning simplification in the medium of causality” (Luhmann, 2005, 97f.; Luhmann, 1990, 2018, 304). The form-determining *difference* results from the specific causal relations that are included *and* the indifference to those (still existing) excluded.^9^ This indifference, therefore, is beneficial for the most part since it is the basic principle of how technology works, and it is simultaneously the cause of adverse consequences. An example is the case of all the carbon combustion technologies that which have been employed successfully for many decades and which operate without regard for the relationship between the transfer of energy within our planetary atmosphere and climate conditions.

**FIGURE 1:**
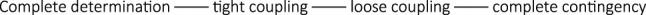
Coupling of events in sociotechnical constellations.

Furthermore, technology must be distinguished from a world of loose couplings. First, technology must be incapsulated from the chaos and complexity of “nature,” the disorganized complexity, and secondly, from “organized complexity” incorporating choice and decision making (Weaver 1948). Organizational theory points to the distinction between a tightly coupled “core technology,” protected from environmental influences, and the loose coupling of events *via* buffering, leveling, forecasting, and rationing (Thompson 1967, 24). Consequently, scholars have argued how organized social systems achieve, on the one hand, tight coupling of operation in order to plan and to recognize deviations from intended operations and, on the other hand, loose coupling as a vital means to cope with surprises, to foster learning, and to increase resilience (Orton and Weick 1990, 205).^10^ We have to assume that sociotechnical constellations are realized in a continuum of different degrees of tight and loose couplings in their operations (as shown in Figure 1).

Yet, we must be careful not to ascribe tight or loose coupling exclusively to either the technical or the social realm. The arguments presented above hint in the direction of attributing social processes the quality of being predominantly loosely coupled. One might assume, because they are the result of selections, and therefore achieve a higher degree of contingency, social relations are exclusively loosely coupled. Highly structured and institutionalized situations might also qualify as tightly coupled, since in such situations not everything is possible and the likelihood of certain, expectable events increases (Schülein 1987). On the other hand, discussions of artificial intelligence explore loose couplings, degrees of freedom, and self-selectivity in technology (Esposito 2017).

In fact, STS research strongly suggests that we have to consider how tight and loose couplings work simultaneously in order to produce expected outputs. Scholars of large technical systems (LTS), for example, have highlighted the functional alignment of meaningful actions and technical operations (based on physical, chemical, and nowadays even biological realities), allowing for the emergence and reproduction of a seamless web (Hughes 1986, 282). In another branch of related research, conceptions of infrastructure systems also focus on entities with both tangible and nontangible elements (Mayntz, 1993, 98). Infrastructures not only entail all kinds of artificial components but also psychological, cognitive, and social elements that are linked to each other recursively on the basis of their functions (Krohn, 1989, 38). In the same vein, actor-network theory investigates many cases where tight (mechanical, hydraulic) and loose couplings (design or user choices) are in effect simultaneously, as in the construction of doors and door-closers (Latour 1988).

− In order to investigate the importance of designating systems, networks, or constellations as being sociotechnical, the above elaborated arguments present us with the guiding distinction: the operationalization of sociotechnical entities emerges as a hybrid construction of simultaneously *tightly coupled* contingency eliminating deterministic occurrences (including automated, routinized human behavior, i.e., *technique*) and *loosely coupled*, contingency evoking, nondeterministic occurrences with a capacity to absorb uncertainty and sustain flexibility in social action and decision making. In all its complexity we arrive at multiple ideal types of how events are aligned to solve functional problems to control, manage, or exploit contingency in sociotechnical constellations (see Table 1):

**TABLE 1:** Tight and loose couplings in sociotechnical entities.

		Operation/activity (occurrences)	
		*technical*	*social*
coupling of events	*tight*	strict determination (the attempt to eliminate contingency)	routine, automated behavior (to restrict contingency)
	*loose*	smart, intelligent, self-diagnostic/healing and learning machines (to allow bounded, controlled contingency)	social communication: choice, decision making, learning (to allow or provoke contingency)

In the following, I will examine how different theoretical approaches describe the composition of tight and loose couplings. The next section will focus on structures of control, how different strands of literature describe them, and how this use of the distinction exposes specific problems for energy transitions.

## Structures of Control in Sociotechnical Operation

### Control in Production, Organization, Networks, and Institutions

The structure of sociotechnical entities evolves in accordance with the purpose of producing a service or output. In order to achieve this, technical systems are designed and social organization is planned according to instrumental operative principles of *conservation* (to prevent change), *transformation* (to enforce change), *storage* (to prevent change of localization), and *transmission* (to enforce change of localization). In complicated sociotechnical entities all these principles take effect simultaneously (Beckman 1994, 320).^11^ Every realization of sociotechnical structures entails tight and loose couplings simultaneously to various degrees. However, instrumental operative principles can only be employed with functioning tight couplings. This also means, loose couplings must be held under control as well.

The challenge here is twofold. First, operators should not be surprised and overwhelmed by unknown tight couplings (Perrow 1984). Secondly, nontechnical operations must be fenced in and made as predictable as possible. This kind of high technology as is employed in power plants demands intelligent organizational design and management for control to be maintained (and safety, too) in tightly coupled organizations confronted with complexity and change (La Porte 1994, 209).^12^ The available historical documentation of accidents highlights the crucial relation between tight couplings in technical operations and the interpretation of data, risky decision making, and acting on incomplete information and under societal, cultural pressure (Vaughan 1996; Higginbotham 2019).

Overall, the desired and planned output of sociotechnical constellations is achieved, as scholars observe it, by utilizing technology and the social organization of production, such as employing personnel, division of labor, differentiation in functional divisions and departments, as well as through cognitive or normative expectations such as scientific and engineering knowledge or norms, laws, and regulations (Mayntz 2009, 123f.). Scholars increasingly use the term sociotechnical to refer to a networked structure of different types of systems and subsystems: “In general […] infrastructures are not systems. Instead, they are networks or webs that enable locally controlled and maintained systems to interoperate more or less seamlessly” (Edwards et al., 2007, 12).^13^ The notion of infrastructures as networks presupposes a mix in the language used in order to operationalize what scholars target in their analysis. On the one hand, we find closed and relatively stable social systems, for example, an organization operating a local power plant, and technical systems such as physical installations and engineering equipment. On the other hand, we find “links” and “nodes” in order to describe how the linking of these systems forms an open and reconfigurable network, such as a power plant that is coupled to a transmission grid with, e.g., transformers and cables, which are controlled by network operators and supervisory organizations *via* nodes. The integration of these networks evolves in the direction of higher level functions and services as a *network of systems* or even a *network of networks*, like internetworks or webs (e.g., electricity markets that are coordinated by price mechanisms). Systems, networks, and webs differ mainly in their access options and the degree of control (Edwards et al., 2007, 12).

Nonetheless, basic instrumental operative principles, as mentioned at the beginning of this section, must also be upheld in network structures in order to maintain a desired output such as provision of energy, transportation, or access and distribution of data. The capacity to compare and adjust actual to intended behavior must grow with the complicatedness or complexity of a given sociotechnical entity (Nightingale et al., 2003). This entails control of the performance of technical components and control of the behavior of persons in their relevant roles such as technicians, workers, engineers, operators, supervisor, and consumers. The skills and knowledge (the *technique*) these roles demand vary in order to be functional and to align to the respective technical operations. Technicians and engineers must know about the complicated causal relations in their field of responsibility.^14^

In addition to such considerations of networks, scholars often refer to institutions or institutionalization. In this case they indicate how events in institutional arrangements are increasingly tightly coupled via commonly shared cognitive and normative knowledge. This entails control of and by the participating parties, aligned via an institutional background, made up by rules to enable corporate actors to legally own, buy, and sell technical commodities, by the rules requiring inclusion of the public in vital services at specific costs, times, and quantity/quality, and by the rules concerning safety, health, and environmental protection (Franssen and Kroes 2009, 224). In the same vein, Künneke et al. (2010) discuss the institutional arrangements necessary to safeguard critical transactions of sociotechnical systems. In a very abstract sense, the concept of institutions is the means to express the possibility of exerting control (including of loose couplings) without maintaining complete determination of events.

### Problems of Control in Changing Sociotechnical Entities

Control is dependent on the emergence, establishment, and sustaining of structures of production, social organization, networks, and institutions. Against this backdrop, research addresses the effect that *change* has *on control* in sociotechnical entities. Mayntz argues that technical development and institutional change are only loosely coupled. They can occur independently, but there is a correlation. For example, the “liberalization” of infrastructure services might have been initiated by a deliberate choice of policies without technical innovation; however, without innovations—especially in coordination technologies—the policies might not have been viable (Mayntz 2009, 144). She addresses the widely acknowledged shift in sociotechnical constellations from

1) Tightly controlled physical structures (centralized production sites, cost-intensive networks) and highly selective access to networks limited to only a few competitors, to
2) The integration of more diverse technical components (wind, solar) and less rigid access that is more accessible to competing parties along the line of production, transmission, and distribution of services.

In abstract terms, the exogenous political program has introduced increased dynamics and complexity to the system via more diverse technical and social elements and varying interrelations (Mayntz 2009, 143).

The dominant theme of recent developments is the *smartification* of control in the energy domain (Lösch and Schneider 2016), the introduction of *artificial intelligence* (AI) (Ali and Choi 2020) and, in the following, the idea of *converging infrastructures* (Büscher, Ornetzeder, and Droste-Franke 2020). Visions of smart grids represent a system transformation in favor of decentralized energy generation with the intention to create an interwoven network of various energy sources, distributive structures, storage capacities, and, significantly, the inclusion of active consumers (Ramchurn et al., 2012). Sector coupling is another issue that in the coming years will have far-reaching consequences in terms of

− *Centralization/decentralization*: If more complicated tight couplings are introduced in future infrastructures, the danger of systemic risk may increase (Hellström 2009, 327). Failures in one part of the overall complex may possibly lead to a cascade of failures in other parts if *sectors* (systems) become more and more *coupled* (Perrow 1984). At the same time, with the emergence of a more decentralized energy system, more redundancies come into effect, which might increase the resilience of the overall complex (Kröger and Nan 2018).
− *Integration/disintegration*: United States and European policy has for decades supported vertical disintegration along the value chain of energy provision, transmission, and distribution (unbundling) to foster greater market-oriented coordination (Coutard 1994). In the case of sector coupling, system planners and supervisors, on the other hand, focus on horizontal integration—*via* the coupling of different infrastructures. This means, for example, that every regulatory attempt must account for the consequences of decision making in a much larger and more complex entity. Planning and risk assessment are thus faced with new problems of responsibility and liability.

These sketchlike descriptions can only hint at problems of control, e.g., reliability, safety, and desired performance. However, we gain an understanding that the relation between “social” and “technical” is not the main analytical focus for these issues. Rather, in the process of an energy transition the shift in the relation between loose and tight couplings is far more consequential. Changing constellations between tightly and loosely coupled social and technical operation will have various effects on planning, control, and governance. The problem of control emphasizes the need to utilize tight couplings—despite increasing complexity—in order to sustain sociotechnical operation. Some of the successful tight couplings in the energy complex (also in, e.g., transportation) pose a problem for sustainable development, and they are hard to get rid of. In the next section, I will discuss conceptual choices concerning the relation between social and technical in *processes of change*.

## Change in and of Sociotechnical Entities

A transition from one sociotechnical configuration, constellation, or system to another affects the patterns of tight and loose coupling. Stimuli for change are attributed in the literature to different factors, such as technological innovation born out of problem-solving inventions, or organizational or institutional innovation co-evolving with the growth of systems, as well as actual or anticipated changes in the environment challenging the basic premises of the reproduction, sustainment, and viability of the system. All these factors interact and influence each other to an (mostly) unknown degree. Change happens all the time, sometimes gradually, sometimes more radically.^15^ Concepts for energy transition refer to more disruptive changes—from one system to another—in the realm of an overall effort to achieve, e.g., the transition of society toward sustainability (Elzen, Geels, and Green 2004; Grin, Rotmans, and Schot 2010; Geels et al., 2012). Transition research therefore assumes that, alongside the relevant technologies, the organizational forms of production and consumption as well as the generalized coordination of action must be changed simultaneously with changes in values and preferences (Rip and Kemp 1998).

While it might be a feasible task to incrementally replace old technologies with new ones, it will be more demanding to change established routines of organizing and coordinating the services around these technologies, or to change users’ routines and practices. The reason is that the now undesirable configurations made large technical systems successful in the first place, as indicated in the previous section. Only tight control through physical and organizational structures can ensure the input–output function of a system and, subsequently, its actual operation.

### Technology as a Limiting or Enabling Factor

In recent years, especially in regard to energy and sustainability transitions, there has been growing awareness that (some) tight couplings can become barriers to change. Once established, tight couplings are hard to get rid of. This topic has been extensively discussed in terms of *path dependencies* ^16^ and of *lock-ins* via embedded organizational practices, routines, and the predominance of increasing returns (Arthur 1989). Also, the phenomenon of *inertia* in systems has been widely discussed (Unruh 2000). In many cases, the problem posed by unlikely change comes down to the question as to what extent technology actually determines the development of sociotechnical relations or, vice versa, how social relations influence technical developments (Rammert 2021). The former thesis has been discussed on a continuum between social constructivism and technological determinism (Dafoe 2015) in various considerations of technology as a factor influencing social development. Such studies include classical works such as on momentum (Hughes 1983, p. 16), technology politics (Joerges 1999), or technological closure and the attribution of meaning (Pinch and Bijker 1984). The second thesis has been looked at, by now, from every conceivable angle, such as social practice (Reckwitz 2002; Shove et al., 2012),^17^ organizations, innovation fields (Markard and Truffer 2008), sectoral change (Dolata 2009), and society. We can relate to the argument by Dafoe (2015, 1,054), who declares technology determinism to be an empirical question of

1) How strongly technologies restrict/support behavior and choice,
2) How far the consequences of investing in, implementing, and using specific technologies are foreseeable, and, consequently,
3) The temporal, spatial, and social context of the relations between the technical and social developments being observed.

Some of the prominent arguments are quite obvious, such as the dynamics of innovation pathways or lock-ins, the overall dependence on a technological base, and the question of to what extent new technologies need to align to already existing ones. Technical determinism originates, for example, in physical causalities, such as maintaining a standard frequency (50/60 Hz) for the transmission of electricity. This requires a constant inflow–outflow rate and, consequently, a set of activities such as economic load dispatch, system structure monitoring, system state measurement and estimation, system security monitoring, load management, load forecasting and generation scheduling, and maintenance scheduling (Amin 2001, 23). In principle, the source of electricity fed into the grid is irrelevant for maintaining the standard frequency. Yet, in a power grid with fluctuating renewable energy sources incorporated, these requirements force system operators to plan with redundancies (back-up power plants) or, in the future, with vast surplus capacities and electricity storage facilities. Some researchers are exploring the possible role of demand side management in adjusting industrial and domestic activities according to the requirements of the power grid.

As a consequence, technology is often discussed as both an enabling force and a limiting factor. Sociotechnical approaches emphasize the very existence of artifacts and technical networks, the interdependencies between components that limit their exchangeability, generalized technical norms and standards, and social activities adapting to and relying on these material premises. The exchange of material elements in a system therefore also requires changes in human behavior and social organization (Shove 2004; Van der Vleuten, 2004). And this is dependent on the possibility of enacting loosely coupled social action and decision making.

### Tight and Loose Coupling at Multiple Levels

Approaches in transition research often make use of the so-called *multilevel perspective* (MLP) to provide a heuristic for understanding sociotechnical configurations.^18^ The MLP concept is attributed to Rip and Kemp (1998) and was refined and further developed by, among many others, (Geels, 2004, 2005). The basic approach analyzes how different constellations of tight and loose couplings can be analytically differentiated (in “levels”) and how these structures are coupled to each other. At the core of the analysis, the authors carve out a construction of highly institutionalized activities in “regimes.” At the mesolevel, Rip and Kemp (1998, 338) refer to the notion of a regime as “the rule-set or grammar embedded in a complex of engineering practices, production process technologies, product characteristics, skills and procedures, ways of handling relevant artefacts and persons, ways of defining problems.” Regimes are generally characterized by a high degree of stability, i.e., structuration (Fuenfschilling and Truffer 2014), where innovations tend to be incremental. This argument refers to the control aspect we discussed earlier. With the terminology used in this paper, it is possible to describe how a regime emerges and maintains itself if it manages to isolate core activities from their environment in the course of fulfilling a societal function (de Haan, 2011). Exogeneous events, therefore, cannot impact regimes one-to-one because they are only *loosely coupled*.

Nonetheless, in the MLP concept every regime is related to and dependent on a loosely coupled “landscape,” which represents the most abstract level in the analysis of transition processes. The landscape refers to the slowly changing environment of the observed regime. As landscapes evolve via natural, macroeconomic, or demographic developments, regimes are similarly affected in specific ways depending on their technology base and cognitive makeup, such as overarching technical and organizations paradigms. However, especially the idea of loosely coupled “niches” raised hopes for a boost in the development and implementation of more sustainable technologies. At the microlevel, scholars are interested in the possibility of decoupling niches from the direct influence of regimes, and therefore, increasing the likelihood of radical innovation (Kemp, Schot, and Hoogma 1998). Innovation takes place in a protected space, because challenging actors operate temporarily without immediately coming into direct competition with the approaches used by incumbents (Geels et al., 2012, 53). Bruns et al. (2010, 389) come to the conclusion that political “niche” development was a decisive factor in the innovation biography of renewable energy technologies. Also, Fuchs (2014) highlights how challenging actors found ways to form a coalition against incumbent actors in the energy complex.

Within the heuristic framework of the MLP, the notion of sociotechnical serves the purpose of shifting the emphasis from problems of control to those of societal change. This is consistent with similar approaches that have been published in recent years. Especially in the case of carbon-dependent combustion technologies, we can experience how technology in isolation works to perfection, and how this success leads to increasing greenhouse gas concentrations in the atmosphere and all other kinds of deterioration of the natural environment. The successful isolation of physical causalities is to blame for the damage, while at the same time recognizing that one cannot abandon this principle after all. Incumbent actors exploit established rigid couplings, protect vested interests and fend off competition for as long as possible (Fuchs 2014; Kungl 2015). At another level, as approaches using MLP claim, new and emerging actors attempt to operate in niches in which they are able to forego some rigid couplings and invent, test, and finally push for the introduction of new ones. However, in exposing unwanted tight couplings, the use of the MLP changes the focus completely to *human activities*, i.e., interactions between various actors and groups in networks, which are guided by structures and institutions and which simultaneously reproduce these structures and institutions.^19^ Therefore, the hope for sociotechnical change feeds on the idea of loosening tight couplings in *social constellations*.

### Deinstitutionalization: Loosen Tight Couplings

In the context of energy transitions, many ideas are being developed that are aimed at ridding society of the previously introduced tight couplings that have led to society’s dependence on certain technologies—certainly and finally in order to disseminate more sustainable technology, for example, energy from renewable sources. Yet almost all proposals fall on the side of *loosening* couplings regarding choices and decision making. Scholars propose unlearning, i.e., to “exnovate” common practices and knowledge in order to learn new ones (Gross and Mautz 2015, 4), to replace dominant “field logics” with more sustainable ones (Fuenfschilling and Truffer 2014), to destabilize regimes (Geels 2014), or to decrease resilience in some parts of the energy domain while increasing it in others (Strunz 2014).

A vast body of literature on transition (or transformation) supports change and refers to the window of opportunity where the promotion of alternatives—differing in their guiding premises from dominant paradigms—is more likely to succeed in “democratic energy politics” (Stirling 2014). Decision premises serve as the pre*condition* for further decisions (not their pre*determination*).^20^ They are taken for granted when used, but do not establish a logical or causal relation. Rather, premises set limits for a multitude of future decisions, and those decisions will be observed in terms of compliance, i.e., conformity or deviation (Luhmann 2018, 181f.). Technology is one important premise for social life; others are, e.g., rules, norms, beliefs, programs, and roles.

In a very broad sense, de Haan and Rotmans (2011, 94f.) argue that “tension, stress, pressure” lead to reflection and deviation from decision premises, i.e., to the realignment of regime–niche relations on three levels: 1) top-down choices by governments about preferred technologies, mechanisms of social coordination (hierarchy, market), or political power shifts among national and supranational entities (such as the EU and its member states); 2) bottom-up empowered alternative niche developments that influence technological choices and forms of cooperation, leading in the end to the institutionalization of alternative norms, values, knowledge, and practices; and 3) internal adaptation of incumbent organizations by rewriting decision programs in order to reproduce (survive) on the basis of deviating fundamental choices, e.g., of business areas (targeting a different customer base), alternative functioning (innovation, new knowledge), or reorganization (growing or shrinking, forming or merging groups, divisions, or specialties).

Scholars of transition research often jump on the bandwagon that politics should choose the right premises in order to absorb uncertainty for all other decision-making activities. For example, Bolton and Foxon (2015, 548) expect policy makers not to continue on the path of reducing the costs of incumbent infrastructures, but to consider transforming systems by, for example, “enabling the integration of renewable technologies, improving energy efficiency and enabling demand side management.” Shifting attention, reducing misalignment among policy levels and sectors, or increasing support for niche activities are reoccurring themes (Negro, Alkemade, and Hekkert 2012). Others propose tackling persistent problems (Schuitmaker 2012) or blocking mechanisms such as uncertainty or the lack of standards and educational programs by supporting the appropriate policy measures (Bergek et al., 2008, 422).

What we must acknowledge is that in ever changing sociotechnical constellations, the structure of tightly and loosely coupled events remains intact. One “configuration that works” (Rip and Kemp 1998, 330) transforms into another configuration. Scholars who use the notion of sociotechnical as a vehicle to address change emphasize loose couplings in their conceptualizations, e.g., decisions leading to political or investment programs to support renewable energy sources (see Table 1). Those decisions might ultimately lead to alterations in tight couplings (e.g., renewable energy technologies). The energy transition, as it proceeds right now, modulates, unbundles, and reconfigures established technological paradigms, vested interests, behavioral routines, and much more, while keeping the output constant, i.e., providing useful energy. Transition research is primarily interested in the alteration of loose couplings and in purposeful, directed societal development. With the focus on loose couplings, assessing the social dynamics resulting from a myriad of micro processes becomes the challenge (Sovacool and Hess 2017). However, since these processes are only loosely coupled, they can hardly be controlled and certainly not enforced. This often leads to a high level of disappointment (Geels, 2015; Raven et al., 2016).

## Rearranging Tight and Loose Couplings in Digital Sociotechnical Realities

### Flat Simplified Screens and Deep Complicated Networks

Sociotechnical entities rely on strict couplings as a necessary means for maintaining control in order to produce an output (e.g., useful energy) on a constant basis. Nonetheless, a strict coupling can become a problem for sustainable development and need be replaced by another. As a consequence, actors operating in sociotechnical entities provoke and exploit such couplings, striving to achieve change by means of technocratic/economic management, policy making, or inclusive, participatory exercises, among other short- and long-term forms of decision making. However, what happens if we encounter loose couplings where strict ones are expected?

More often than not, technological networks are an invisible part of our social existence (Edwards et al., 2007). Yet technical operations and human activities—although mutually deeply embedded—take the form of different *modi operandi*. They are interdependent and tight and loose couplings in effect simultaneously. This does not correspond to the network character of the seamless web; it rather introduces the notion of “orthogonality,” i.e., the concomitant and continuous reproduction of technical output and social occurrences.^21^ Each can only affect the other sporadically and then only temporarily. On a regular basis, action and decision making fulfill the purpose of monitoring or controlling technical processes in terms of implementing, testing, adjusting, and observing operation. In other cases, unforeseen and unplanned strict couplings sometimes cause disturbances or accidents that provoke contingency and crisis management.

Luhmann considers technical networks, especially those providing the continuous flow of energy, as neutral to communication because all information is derived in a communicative process of social action and in cognitive processes of consciousness (Luhmann, 2012, 180). The ongoing process of digitalization in particular raises new questions as to the relation between tight and loose couplings. Despite the tremendous development in data processing capacities in terms of speed, volume, and accessibility, algorithms themselves do not produce contingency—they process tightly coupled operations. Additionally, we are still dependent on interpretation and choice. However, as Esposito plausibly claims, the interaction of humans with “smart technology” and forms of “artificial intelligence” might lead to surprises and learning: “People learning to learn from machines increases the complexity of communication in general” (Esposito 2017, 262).

The control of large technical systems has always posed a challenge. Yet, if we look at modern, digitized networks for example, the world-wide web, power grids or aviation control, the ongoing data processing and the assessment of results have further emphasized the distinction between “deep” and complicated structures and their “flat” representation. Operators rely on models of the physical networks displayed on screens (previously analog operator boards). Signs, symbols, and signals have to be put into relation to the state of the net in reality, which is not assessable in its entirety. It is only through expert knowledge that the interpretation of the data is possible. Operators and users of digital infrastructures need to cope with the relation of *flat* computer screens (representing the results of the model calculation) with the *deep* and complicated (physical and electrical) structure of the system behind the model. New dissemination media provide tremendous computing speed and the capacity to store vast amounts of data, which is being permanently updated and replenished by semantic webs and social media. This data is made accessible by elaborate search algorithms and interconnects human activities (Baecker 2007, 24) in an internet of things (IoT), internet of people (IoP), industrial internet (II), or internet of everything (IoE) (Demirkan et al., 2015). As this development demonstrates, the rift between simple interfaces and deep technical networks, between face-to-face interaction and anonymous communication, and between personal contacts and interaction with machines is becoming more and more thorough. In essence, the tools (algorithms and software) for overcoming the cognitive limits of humans are generating a new form of nontransparency where operators, supervisors/regulators, or end users cannot fathom all the elements and relations between these elements any more.

### Introducing Loose Couplings in Deterministic Operation

This line of argumentation gives rise to specific research problems in the energy nexus. The current debate about the transformation of the energy infrastructure includes far-reaching visions (expectation statements) which go beyond the currently prevailing paradigm of automation. The power supply should, in the course of the energy transition, become increasingly reliant on the fluctuating energy sources solar and wind. New technology, mostly ICT-related innovations, is in the process of deployment in order to support better control of the associated fluctuations in supply. This technology is advertised as featuring such properties as “autonomy” and “artificial intelligence” (e.g., smart, intelligent, and self-healing) (Ramchurn et al., 2012; Kakran and Chanana 2018; Ali and Choi 2020). In many visionary concepts, this kind of technology should moreover be deployable by the electricity purchaser. A prerequisite for this is a two-way exchange of data between providers and purchasers of electricity. In the background, intensive data analysis is necessary in order to generate information in real time about user behavior, on the one hand, and about prices, on the other (for a summary see Lösch and Schneider, 2016).

For these visions to become reality, technical innovations must be accompanied by a redesign of the means to coordinate behavior. For the transformation of the energy supply to succeed as a major social project,

− operators, managers, regulators, and legislators have to cope with an ever increasing sociotechnical complex made up of a network of heterogeneous elements that interact with the support of digital machines (Kröger 2017).
− a significant part of the affected population may have to take on a new role, namely that of active consumers (prosumers) in digital networks coordinating supply and demand; they at least are supposed to utilize smart technology, for example, in their homes, at their workplaces, and when traveling (Verbong, Beemsterboer, and Sengers 2013).

These prerequisites must be achieved by means of innovative market models and/or new legislation. Yet these mechanisms alone are not enough. It is not only the immense increases in the speed, storage, and interconnectivity of computational capacities that place a burden on the communicative capacities of human beings and, for that matter, of organizations (Wildavsky 1983) to cope with an overflow of data, information, and knowledge. It is also the degree of opaqueness of the immense technical networks that increases uncertainty about who is collecting data and to what end; who is intruding and watching without consent; and what is a trustworthy source of information.^22^ This is based on a confluence of complicated (or already complex?) technical and social event linkages. We are used to coping with double contingency in social interactions, but not yet in dealing with technical devices and software.

### Social Arrangements to Cope With Contingency

While loose couplings are necessary for change—such as degree of freedom, wiggle room, or an opportunity for learning, creativity, or challenges—emphasizing loose couplings in sociotechnical constellations raises awareness for problems of contingency, complexity, uncertainty, and risk, especially in regard to decision making (see Table 1 for a comparison). Researchers are challenged, then, to investigate how the constellation nonetheless functions, what the points of friction are, and how they might possibly be removed. Within the framework of the proposed energy sustainability transformation, the generalized coordination of action under uncertainty is important. In this sense, social mechanisms of authority and trust are crucial and functionally equivalent to such symbolically generalized mechanisms as money, power, law, and knowledge (Büscher and Sumpf 2015; Sumpf, 2019). Solutions to problems of contingency, complexity, uncertainty, and risk are subject to different conditions. In the case of local conflicts over *siting decisions*, the interaction between decision makers and those affected comes into focus (Dwyer and Bidwell 2019). For those affected by these decisions, acceptance can constitute an important moment regarding an understanding of the opportunities (efficiency, sustainability) or the risks (dangers to health, property, aesthetics, or else) (Kasperson and Ram 2013). However, this is not the problem in focus here if we look at a large-scale transition driven by *digitalization*. At focus is rather the issue that action capacity is crucial for individual actors who want to participate—and who therefore have to make decisions—in determining modern forms of energy provision, i.e., a so-called prosumer, whether concerning virtual power plants or community energy initiatives (Seyfang, Park, and Smith 2013) or the use of smart home appliances (Hansen et al., 2020; Patterson-Hann and Watson 2021), for example.

Also crucial in terms of enacting a program for the transformation of existing systems or of a network of systems is the degree of uncertainty that operators, regulators, and managers have to cope with as well as the uncertainty of those who have to sustain the transition to a more sustainable energy infrastructure, such as an innovative policy maker. All the above-mentioned participants, and many more, will be incorporated in a vast sociotechnical network. Uncertainties at the level of individual and organizational decision making can lead to blockages, with the consequence that individual actors refrain from participating, companies from investing (or worse), and administrators from undertaking necessary action in crisis situations. At the level of the overall complex, this might undermine a generalized exploitation of opportunities with regard to the energy transition. In such a context, the often applied focus on the “generation” of acceptance is not enough. Rather, one has to assume that the interaction of (latent) confidence in the viability and chances of success of the overall transition project and trust in systems (as impersonal trust) can generate a capacity for action (Sumpf, 2019).

How energy transitions affect social systems depends on their structural disposition. As an object of economics, politics, science, law, and more, transitions evolve without the guiding hand of a central influence. Transitions, accordingly, are treated differently in the subsequent functional systems of society, which plan, strategize, and operate along their respective rationalities and do so simultaneously. Therefore, humans are used to nontransparency in social interaction with persons, in dealings with organizations, or in their expectations toward entities like markets. The digital world, which is now increasingly entering basic infrastructures, introduces another level of loose couplings (along with contingency) into the experience of sociotechnical entities. Operators, supervisors, and users have to cope with the opaqueness arising from this constellation and need to develop means to continue to be able to act faithfully (Baecker 2007).

## Conclusion

The notion of sociotechnical generates added value by pointing to the relevance of the interrelation between technical and social realities in fields like energy transition, energy system transformation, or in a broader sense societal change in the direction of more sustainable development. The term sociotechnical, however, often remains quite underspecified along expressions like social and technical elements are “aligned” or the technical is “embedded” in the social. This contribution makes the case for exposing “tight” and “loose” couplings of operations and activities as the distinctive feature of sociotechnical entities. In this respect, all of the research directions re-interpreted here can offer substantial analyses in different ways. This depends on whether the emphasis of observation is on couplings that allow for control (tightly coupled technical and individual/organizational/societal action) but are—in its current configuration—not sustainable, or whether it is on possibilities for change that are fostered by exploiting loose couplings, which can take the form of more desirable decision premises. Novel avenues for further curiosity open up with the perspective of loose couplings introduced in the technical realm, e.g., in form of smartness and intelligence.

The object of research—energy infrastructures or large technical systems—can be understood as a complex which is the focus of generalized expectations towards services and outputs, and in which a variety of different units participate and contribute to fulfilling functions (providing and receiving services). The way in which the relationships between the social and technical units are described depends on the purpose of the research and the problem being addressed. It is only then that we see

− that tight couplings are necessary for maintaining control despite the increasing complexity we observe right now; and that new forms of social coordination and organization will be necessary to cope with smart technologies, coupled infrastructures, and autonomous machines;
− that some of the tight couplings are hard to get rid of. This is not only because of idiosyncratic interests, decoupled from any ideas of common goods, but also because social relations are dependent on stable orientation (in which familiar technology plays its part as well). Therefore, instigating change despite the need for stability demands many forms of creative ingenuity, a sense for experiments, and risk taking;
− that all kinds of tight coupling that we nowadays depend on create an asymmetric relationship between simple interfaces and “invisible,” yet very complicated architectures. Therefore, achieving and maintaining the capacity to act and to make decisions despite nontransparency demands trust and confidence (for example in the capabilities and goals of systems) to actively pursue the manifold opportunities of a sustainable energy transition. This is especially true if we enrich these networks with smart, intelligent, and self-learning machines, which as “non-trivial machines” (von Foerster) generate their own history.

With a phrase coined by Paul (Edwards, 2004, 209), these issues can be framed as continuous “sociotechnical problems.” As a common point of reference, sociotechnical problems might constitute a useful substitute for the peril of generating a shared understanding of the system in focus, i.e., a standardization of models, constructs, or terms. Yet this argument has to be unfolded elsewhere (Büscher 2018).

## Data Availability

The original contributions presented in the study are included in the article/Supplementary Material, further inquiries can be directed to the corresponding author.

## Footnotes

1 By employing the notion of SSH energy research, we align ourselves with an interdisciplinary field that has gained momentum in recent years, such as on the European level (see https://shapeenergy.eu/index.php/about).

2 Once in the introduction and more explanatory in the epilog: “Such encompassing systems should be labeled sociotechnical systems rather than technological systems” (Hughes 1983, 465).

3 See, for example, in Innovation Research (Markard and Truffer 2008), Social Practice Theory (Shove et al., 2012) and many more approaches summarized in Sovacool and Hess (2017).

4 The emergence of innovations in sociotechnical entities, e.g., new practices, new business models, new technologies, and new forms of policy intervention, is affected by the application context, actor constellations, and geographic areas with their specific characteristics (Raven et al., 2016).

5 In this regard, I follow the theoretical premises of “operative constructivism” (Luhmann 1990, 49; Luhmann 2018, 300f.) See for theoretically similar arguments in practice theory Reckwitz (2002) or in transition theory: “Actors reproduce the elements and linkages in ST systems in their activities” (Geels 2004, 902).

6 See (La Porte, 1975, 6) for a definition of complexity relating to the problem of selective relationships between elements.

7 In a very abstract sense, I will refer to the term “contingency” as something neither impossible nor necessarily inevitable. If something is designated as contingent, it is deemed possible to be otherwise (Luhmann 1995, 25). This term is of utmost importance, because contingency refers to the possibility of other information, other outcomes of decisions, and in this sense, to alternative decisions all together. Contingency, therefore, is related to *risk* and *uncertainty*.

8 Wind energy converters, for example, use thermic causalities as a force to propel large rotor blades coupled to power generators. Engineers and operators are able to control specific mechanical causalities, without controlling others such as atmospheric conditions. The latter causalities are not under the system’s control, but still influence its behavior. They are part of the environment and, therefore, operators are dependent on predicting environmental factors to maintain control.

9 This characterization can be found also in philosophical writings, like: “Technology in essence pertains to the realization of clear goals, which are realized through appropriate measures that systematically eliminate all disturbing influences” (Rapp 1987, 115; my translation).

10 In social theory classical authors have emphasized the selectivity of social relations, for example: replacing instinct-based responses to offers of interaction with yes-or-no selection (Mead 2009); meaning selections in action (Weber 1978); selective adaptation in social situations in order to establish affiliation (Schütz 1944); the problem of structured double contingency (Parsons 1991); the continuation of communication as a synthesis of a threefold selection of utterance, information, and understanding (Luhmann 1995, 140ff.).

11 For example, in facilities like nuclear power plants designed transformations must be organized in strictly closed containments in order to generate and transmit the intended output: Nuclear fission releases a large amount of energy to heat a coolant. The coolant is pumped through the reactor, transmitting energy away from the reactor in order to transform the state of such coolant from fluid to gaseous (steam). The steam runs through high- and low-pressure turbines, which in turn transmit kinetic energy to generators. The generator transforms the kinetic energy into electrical energy. Large parts of the operative cycle are kept from interacting with the environment by being enclosed in a concrete chamber.

12 For comprehensive discussions about the conditions and limits of reliability, control, and safety in complex sociotechnical constellations, see (Sagan, 1993; Sagan, 1994), Perrow (1984; 1994), and (La Porte 1994, 1982).

13
Edwards et al. (2007) look especially at “cyber” infrastructures.

14 Contrary to most consumers who cope with interfaces and basic rules of behavior and otherwise do not have to engage in the intricacies of the service production. See an early account from Schütz (1944, 501): “Man in his daily life is only partially […] interested in the […] full insight into the relations between the elements of his world and the general principles ruling those relations.”

15 We recognize change only in hindsight, we cannot recognize it while it happens, and only with the help of earlier reports, such as studies by (Fouquet, 2009; 2016) or Ayres and Voudouris (2014).

16 Widespread technical norms or physical structures that cannot be replaced in a short period of time, such as for railroads, sewers, road systems, or transmission and distribution lines for electricity. See, e.g., Urry (2004).

17 “As elements link to form practices, so practices connect to form regular patterns, some only loosely associated, others more tightly bound” (Shove et al., 2012, 17).

18 The concept of technological innovation systems (see, for example, Hekkert et al., 2007; Markard and Truffer 2008) could also have been presented here. For reasons of space, our focus is limited to the MLP approach.

19 See also Bolton and Foxon (2015, 539), who lose interest in technology early on in their work: “[The] transformation of technologies and technical systems is not determined by any scientific, technological or economic rationality, rather there are a wide range of social, political and institutional factors which interact in a systemic fashion to influence their development.”

20 With reference to individual behavior the concept of “premises” can be found in the writings of Simon, (1959, 274).

21 For which both need a continuous supply of energy (Fouquet 2009).

22 See in reference to the general topic of privacy and data control (Pentland, 2014, 177ff.), Pasquale (2015), or Schneier (2015).
